# Antenatal corticosteroids for early preterm birth: implementation strategy lessons from the WHO ACTION-I trial

**DOI:** 10.1186/s12961-022-00941-z

**Published:** 2022-12-28

**Authors:** Ayesha De Costa, Olufemi T. Oladapo, Shuchita Gupta, Anayda Portela, Joshua P. Vogel, Joao Paulo Souza, Suman Rao, Nicole Minckas, Özge Tuncalp, Rajiv Bahl, Fernando Althabe

**Affiliations:** 1 Department of Maternal, Newborn, Child, Adolescent Health, and Ageing, World Health Organization, 20 Avenue Appia, Geneva, Switzerland; 2 UNDP/UNFPA/UNICEF/WHO/World Bank Special Programme of Research, Development and Research Training in Human Reproduction (HRP), Department of Sexual and Reproductive Health and Research, World Health Organization, 20 Avenue Appia, Geneva, Switzerland; 3 Maternal, Child and Adolescent Health Program, Burnet Institute, The Macfarlane Burnet Institute for Medical Research and Public Health Ltd, 85 Commercial Road, Melbourne, VIC 3004 Australia

**Keywords:** Preterm birth, Neonatal mortality, Antenatal corticosteroids

## Abstract

The WHO ACTION-I trial, the largest placebo-controlled trial on antenatal corticosteroids (ACS) efficacy and safety to date, reaffirmed the benefits of ACS on mortality reduction among early preterm newborns in low-income settings. We discuss here lessons learned from ACTION-I trial that are relevant to a strategy for ACS implementation to optimize impact. Key elements included (i) gestational age dating by ultrasound (ii) application of appropriate selection criteria by trained obstetric physicians to identify women with a likelihood of preterm birth for ACS administration; and (iii) provision of a minimum package of care for preterm newborns in facilities. This strategy accurately identified a large proportion of women who eventually gave birth preterm, and resulted in a 16% reduction in neonatal mortality from ACS use. Policy-makers, programme managers and clinicians are encouraged to consider this implementation strategy to effectively scale and harness the benefits of ACS in saving preterm newborn lives.

## Introduction

Preterm birth, defined as birth before 37 weeks of gestation, is the single largest cause of neonatal mortality globally [1]. Among the most beneficial antenatal interventions to improve neonatal outcomes when preterm birth is inevitable is the administration of antenatal corticosteroids (ACS) to women at risk [1]. ACS have long been promoted as a key intervention to reduce mortality and morbidity from preterm birth, largely on the basis of trials conducted in high-resource countries. The generalizability of this intervention to low-resource settings was deemed uncertain following the results of the large population-based Antenatal Corticosteroids in low- and middle-income countries [2]. ACT, which was conducted in six low-resource countries, showed that efforts to scale up the use of ACS could lead to harm—for both the mother (higher infection rates) and newborn (higher mortality). These findings reopened the debate about the safety and efficacy of ACS in low-resource countries, where there is higher burden of infection and different standards of perinatal healthcare.

The WHO-coordinated Antenatal Corticosteroids for Improving Outcomes in Preterm Newborns (ACTION)-I trial [3] was specifically designed to clarify the uncertainties surrounding the safety and efficacy of ACS use in the early preterm period in low-resource countries [4]. ACTION-I was a hospital-based, double-blind, placebo-controlled randomized trial conducted in five low-resource countries (Bangladesh, India, Kenya, Nigeria and Pakistan) to evaluate whether the administration of a course of intramuscular (IM) dexamethasone (6 mg every 12 hours [q12h] × four doses or until birth, whichever was earlier) to women who were at risk for early preterm birth (26 to 33 weeks 6 days of gestation) improved neonatal survival and was safe for women and newborns. The trial randomized 2852 women (and their 3070 babies) and showed that, compared with placebo, the use of dexamethasone resulted in a 16% reduction in neonatal deaths (relative risk [RR] 0.84; 95% confidence interval [CI] 0.72–0.97), 12% reduction in stillbirth or neonatal death (RR 0.88; 95% CI 0.78–0.99) and no significant differences in maternal bacterial infections. Dexamethasone also resulted in significantly lower risk of early neonatal death, severe respiratory distress at 24 hours after birth and need for neonatal resuscitation, providing further support for the overall reduction in neonatal mortality in these settings.

The findings of the ACTION-I trial—the largest placebo-controlled trial on ACS efficacy and safety to date—are reassuring. However, effective translation of these findings into routine clinical practice in low-resource countries requires several measures to ensure that ACS are used safely to harness their clear benefits. The positive results of the ACTION-I trial underscore the importance of using a similar strategy to obtain the benefits and avoid potential harms of ACS, particularly when used in resource-limited countries.

The aim of this article is to share key information on the strategy that was implemented in the ACTION-I trial [3]. This approach can guide the implementation of ACS in clinical settings, as well as inform future implementation research to safely scale up ACS use.

## Implementation considerations in the WHO ACTION-I trial

### Characteristics of hospitals that participated in the trial

The 29 hospitals that participated in the ACTION-I trial were those that could reasonably meet the WHO ACS administration criteria as defined in Box 1. These were secondary- or tertiary-level hospitals in low- and low–middle-income countries that could provide a minimum package of care for pregnant women at risk of preterm birth and their preterm newborns.

### Minimum package of care for pregnant women at risk of preterm birth

All participating hospitals had the capacity to provide adequate obstetric care including the diagnosis and safe management of preterm labour and birth. At a minimum, each hospital was able to provide the following interventions: ultrasound examinations for gestational age (GA) assessment, parenteral antibiotics for treating peripartum infections, parenteral anticonvulsants for treatment of pre-eclampsia and eclampsia, parenteral oxytocic drugs for postpartum haemorrhage prevention and treatment, manual removal of retained products, blood transfusion, caesarean section and hysterectomy.

Prior to the start of the trial, facilities to routinely perform ultrasound examinations for confirmation of GA were not consistently available in the selected hospitals across the five countries. The ability to perform dating ultrasounds at each facility was strengthened with the supply of ultrasound machines (Philips HD5 ultrasound machine, with transabdominal, transcranial and intravaginal probes), as well as staff training and monitoring to performing GA dating ultrasound examination.

Additionally, hospitals were encouraged to follow the WHO 2015 recommendations on interventions to improve preterm birth outcomes with regard to the use of tocolytics and magnesium sulphate for foetal neuroprotection, and use of antibiotics for preterm prelabour rupture of membranes (PPROM) [1]. However, this was not mandatory, and hospitals were allowed to follow local protocols.

Box 1: Prerequisites for ACS administration according to WHO recommendation [1] Antenatal corticosteroid therapy is recommended for women at risk of preterm birth from 24 to 34 weeks of gestation when the following conditions are met:

- GA assessment can be accurately undertaken;
- preterm birth is considered imminent;
- there is no clinical evidence of maternal infection;
- adequate childbirth care is available (including the capacity to recognize and safely manage preterm labour and birth);
- the preterm newborn can receive adequate care if needed (including resuscitation, thermal care, feeding support, infection treatment and safe oxygen use).

### Minimum package of care for preterm newborns

All participating hospitals were supported to ensure that preterm newborns could receive a minimum package of care, including the following interventions:

- *Care at birth and newborn resuscitation in the birthing room:* Facilities in the ACTION-I trial had a newborn care corner in the labour room with a radiant warmer. All personnel were trained in essential newborn care [5] including neonatal resuscitation using a bag and mask. Functioning equipment for bag-mask ventilation were available, including sizes appropriate for preterm babies.
- *Identification and management of respiratory distress, safe use of oxygen and respiratory support including optimal use of continuous positive airway pressure (CPAP)*: Neonatal care units in all hospitals were supported with CPAP machines to provide noninvasive respiratory support and pulse oximeters to monitor oxygen saturation. Research staff were trained to monitor respiratory rate and identify signs of respiratory distress including chest indrawing and grunting.
- *Thermal care*: Facilities had radiant warmers or incubators to ensure preterm babies were protected from the risk of hypothermia. Staff were trained to monitor temperature at set intervals, and to detect and correct hypothermia by placing the baby under a radiant warmer.
- *Breastfeeding and assisted feeding*: Staff encouraged mothers whose preterm babies were in the neonatal care unit, to breastfeed the newborn or to express breast milk (fed to the newborn by gavage/cup). Early feeding and escalation of feeds was encouraged to limit the need for intravenous fluids.
- *Monitoring of hypoglycaemia*: Staff was trained to monitor blood sugar at 6 and 36 hours of life for timely detection and correction of hypoglycaemic episodes.
- *Prevention and management of infection*: There was an emphasis on infection prevention and control including handwashing on entering the neonatal care unit, sanitizing hands between contact with babies and encouraging the use of disposable goods wherever possible. Staff were trained to recognize signs of infection and possible sepsis. Antibiotics stewardship in the neonatal unit was encouraged.

Additionally, kangaroo mother care (KMC) was encouraged for all preterm and low-birth-weight babies. Special KMC rooms adjacent to the neonatal care unit were available to provide this care in some of the participating hospitals.

It must be noted that neither surfactant nor mechanical ventilation was available for the management of preterm infants in most hospitals, and neither of these was part of mandatory respiratory support provided in the trial.

### Strategy to identify women eligible for ACS administration

#### Assessment of GA

All women considered eligible for the ACTION-I trial had a GA between 26 weeks 0 days and 33 weeks 6 days, confirmed by an ultrasound of sufficient quality during pregnancy. “Sufficient quality” implied that an ultrasound was performed by the hospital sonology service or a reputable ultrasound provider in another facility or health service, and reported foetal measurements allowing for an accurate GA estimation. If a dating ultrasound had not been performed earlier in pregnancy (or was not of reasonable quality based on the judgement of the attending obstetric physician), an ultrasound was performed upon the woman’s presentation at the trial hospital. Besides the provision of ultrasound machines, trial hospital staff involved in performing obstetric ultrasounds underwent a standardized, competency-based training conducted by expert trainers from the International Society of Ultrasound in Obstetrics and Gynecology. A final assessment of GA at trial entry was made using a combination of ultrasound dating and a certain last normal menstrual period (LMP) (Box 2), based on the American College of Obstetricians and Gynecologists algorithm (Table 1) [6].

**Table 1:** Using ultrasound and LMP to determine ACTION trial estimate of GA [6]

	Timing of earliest ultrasound to estimate GA (by LMP)					
	First trimester		Second trimester			Third trimester
	Up to 8 weeks 6 days	9 weeks 0 days to 13 weeks 6 days	14 weeks 0 days to 15 weeks 6 days	16 weeks 0 days to 21 weeks 6 days (by LMP)	22 weeks 0 days to 27 weeks 6 days	28 weeks 0 days and beyond
Method of measurement on ultrasound	CRL		BPD, HC, AC, FL^a,b^			
LMP is certain	If difference is ± 5 days, use LMP If difference > 5 days, use U/S	If difference is ± 7 days, use LMP If difference > 7 days, use U/S		If difference is ± 10 days, use LMP If difference > 10 days, use U/S	If difference is ± 14 days, use LMP If difference > 14 days, use U/S	If difference is ± 21 days, use LMP If difference > 21 days, use U/S^c^
LMP is uncertain or unknown	Use U/S based on careful clinical judgment					

*LMP* last menstrual period, *CRL* crown–rump length, *BPD* biparietal diameter, *HC* head circumference, *AC* abdominal circumference, *FL* femur length, *U/S* ultrasound

^a^Transcerebellar diameter (TCD) can also be optionally used, from 14 weeks 0 days to 27 weeks 6 days

^b^If an earlier dating U/S is available, and is consistent with LMP, use the GA from the earlier scan

^c^If no earlier scan, check TCD, distal femoral, proximal tibial and proximal humeral epiphyses in addition to biometry

Box 2. GA assessment among women enrolled in the ACTION-I trial **In the ACTION-I trial:**

- 100% of the women enrolled had an ultrasound for GA dating: around 10.5% in first trimester, 23.6% in second trimester and 65.7% in third trimester.
- Ultrasound-derived GA was used in those 40% of women who reported unknown or uncertain last menstrual period (LMP).
- The median GA at birth was 33 weeks (interquartile range [IQR] 31–34).

#### Diagnosis of imminent preterm birth

For the purpose of ACS administration, imminent preterm birth was defined as “birth planned or expected in the next 48 hours”. This included situations in which one or more of the following were present (Box 3):

1. Women presenting with signs of spontaneous preterm labour, in whom the likelihood of birth in the next 48 hours was assessed to be high by an obstetric physician, including women with at least six regular uterine contractions in 60 minutes and women with either a cervical dilatation of ≥ 3 cm or 75% cervical effacement.
2. Spontaneous PPROM was confirmed by visible leakage of fluid from the cervix or pooling of fluid in the vaginal vault.
3. Provider-initiated preterm birth for any medical or obstetric (foetal or maternal) indication, in the next 48 hours after administration of ACS, by either induction or caesarean birth.

Box 3. Characteristics of randomized women at time of entry into the WHO ACTION-I trial **In the WHO ACTION-I trial:**

Of the 2852 women randomized:

- 30% had PPROM,
- 31% spontaneous preterm labour,
- 39% a provider-initiated preterm birth,
- 91% had a singleton pregnancy and 9% had a multiple pregnancy.

#### Exclusion of women with infections

Women were not eligible to receive dexamethasone or placebo if there were clinical signs of severe, acute infection. This assessment was made by an obstetric care physician based on the presence of maternal fever ≥ 38.0 C, maternal and/or foetal tachycardia, purulent or foul-smelling vaginal discharge, uterine tenderness, maternal leukocytosis or bacterial culture indicative of infection.

### ACTION-I trial ACS regimen

The ACS regimen included in the ACTION-I trial was 6 mg IM dexamethasone sodium phosphate q12h, to a total of four doses (0 h, 12 h, 24 h and 36 h) until course completion, discharge or birth (whichever came first). If the woman did not give birth within 7 complete days after the first dose, a second identical course according to the regimen described above was administered. This ACS regimen was consistent with the WHO recommendation [1]. Box 4 indicates the ACS dose-to-delivery interval in the ACTION-I trial. Only 57% of women received four doses of first ACS course. The most common reason that a full ACS course was not administered was the occurrence of birth before the time that the fourth dose was due.

Although WHO recommends the use of either IM dexamethasone or IM betamethasone, the ACTION-I trial used IM dexamethasone in view of its wide availability, lower cost and inclusion on the WHO essential medicines list [7].

Box 4. First ACS dose-to-delivery interval in the ACTION-I trial Of the live births of women receiving dexamethasone in the ACTION-I trial:

- 27.0% occurred within 12 hours;
- 35.6% occurred within 24 hours;
- 69.3% occurred within 7 days.

Numbers of dexamethasone doses received by the women assigned to the ACS arm were as follows:

- 57.0% of the women received four doses;
- 62.9% of the women received at least three doses;
- 73.2% of the women received at least two doses;
- 99.9% received at least one dose;
- 4.3% received at least one dose of a second course.

### Scaling up ACS in low-resource countries

The WHO ACTION-I trial provided clear evidence of the beneficial effects of ACS administered in the early preterm period in improving neonatal outcomes among newborns in low-resource settings. The benefits of ACS with respect to neonatal mortality and morbidity in the context of ACTION-I were possibly mediated by the implementation of a strategy that ensured (i) GA dating by ultrasound, as early as possible or at least a confirmatory ultrasound at admission when early ultrasound was not available or reliable, (ii) application of appropriate selection criteria by trained obstetric physicians to identify women with a likelihood of preterm birth and eligible for ACS administration, and (iii) the ability of facilities to provide a minimum package of neonatal care for preterm infants. These elements are key as they allow better “selection” of women who are most likely to benefit (implementation of this strategy resulted in 90% of identified women giving birth preterm i.e. < 37 weeks), thereby minimizing the risk of overuse and possible harm [2, 8, 9]. Further, the availability of a minimum package of care for preterm newborns ensured that once these women gave birth, the newborns received vital care. ACTION-I trial results demonstrate that when ACS is used according to this approach in reasonably equipped secondary- or tertiary-level hospitals in low-resource settings, benefits (and absence of harms) can be assured.

However, there are still many uncertainties to clarify before an ACS implementation strategy can be developed for routine practice at a population level in such settings. The ACS implementation strategy described above was used in the context of a clinical trial. It is still unclear whether the optimal strategy for identifying women who are likely to give birth in the early preterm period would be different in low-resource settings, but it is critical to identify a strategy that works to correctly detect a significant proportion of these women, to enable high coverage of appropriate ACS use at scale. Even under controlled conditions as in the ACTION-I trial, a substantial proportion of eligible women could not receive their full course of ACS largely as a result of giving birth before the scheduled time for a full course. This relates to the inherent inability of the currently available methods to accurately predict time to delivery in women considered eligible for ACS, which is one of the key limitations to effective scale-up. It does emphasize the need for concerted efforts to support healthcare providers in optimizing the methods for identifying eligible women, including consideration for combination of antenatal tests that are likely to improve clinical prediction [10].

We need to learn how to best embed the ACTION-I trial strategy for ACS use in routine health systems at scale in low-resource settings to achieve high coverage of appropriate ACS use in order to reduce mortality and morbidity among preterm newborns.

Current ACS coverage in low-resource settings (~40%), which have the largest share of preterm births, is much lower than in high-income countries (90%) [11]. Scaling up ACS use in routine health systems implies health system strengthening that prioritizes improving access to ultrasound for GA dating for all women, as re-emphasized by the recent 2022 WHO recommendation [12] establishing clear criteria and protocols by which women are identified for ACS administration, as well as ensuring that the necessary maternal and preterm newborn care are available at facilities attending preterm births. Without such measures, harmful effects—such as the use of ACS in women with clinical infections or exposing term infants to unnecessary ACS and the associated risks [8, 9]—may increase. WHO is therefore planning implementation research projects in a number of low-resource countries to generate evidence on the optimal implementation strategies to increase ACS coverage at scale, while adhering to treatment criteria that would optimize outcomes for women and their babies.

## Conclusion

ACS is effective at reducing newborn mortality and morbidity from preterm birth in low-resource settings, when given in line with the WHO recommendations on ACS for improving preterm birth outcomes [12]. The ACTION-I trial emphasizes the importance of carefully selecting women who are most likely to give birth preterm to receive ACS. Widening coverage without these precautions makes both women and their newborns vulnerable to immediate and longer-term risks of ACS. More research is needed to scale up ACS at the population level in low-resource settings, particularly on the optimal implementation strategies that can increase ACS coverage effectively and safely. Until that evidence is available, policy-makers, programme managers and healthcare providers in resource-limited settings wishing to focus on implementing ACS to reduce newborn mortality and morbidity among preterm births are encouraged to consider the implementation strategy described above, to effectively harness the benefits and avoid the potential harms of ACS.
